# Cross-sectional observational study on prevalence and pattern of multimorbidity and its impact on geriatric outpatients in a south Indian tertiary hospital

**DOI:** 10.3389/fpubh.2026.1778787

**Published:** 2026-05-13

**Authors:** Shilpa Avarebeel, Michael Vassallo, H. Basavanagowdappa, Pratibha Pereira, Deepthi Puttegowda, Murali Krishna, Stavelin Abhinandithe K, Ramith Ramu, Mudassar Shahid

**Affiliations:** 1 Department of Geriatrics, JSS Medical College & Hospital, JSS Academy of Higher Education & Research, Mysuru, Karnataka, India; 2 University Hospitals Dorset, Royal Bournemouth Hospital, Bournemouth University, Bournemouth, United Kingdom; 3 Department of Medicine, JSS Medical College & Hospital, JSS Academy of Higher Education & Research, Mysuru, Karnataka, India; 4 Department of Biotechnology & Bioinformatics, JSS Academy of Higher Education & Research, School of Life Sciences, Mysuru, Karnataka, India; 5 Institute of Public Health, Bangalore, Karnataka, India; 6 Division of Medical Statistics, School of Life Sciences, JSS Academy of Higher Education & Research, Mysuru, Karnataka, India; 7 Department of Pharmaceutics, College of Pharmacy, King Saud University, Riyadh, Saudi Arabia

**Keywords:** chronic diseases, cluster analysis, geriatrics, multimorbidity, socio-demographic factors

## Abstract

**Background:**

The global aging population is increasing rapidly, with a significant rise anticipated in individuals aged 60 years and above over the coming decades. This demographic transition is expected to amplify the burden of chronic diseases and multimorbidity, especially in low- and middle-income countries like India.

**Methods:**

This observational cross-sectional study was conducted from November 2022 to November 2024 at a tertiary care hospital in South India. A total of 541 older adults (mean age 71.56 ± 7.06 years; 280 men and 261 women) attending a geriatric outpatient clinic were enrolled. A Comprehensive Geriatric Assessment (CGA) was performed to capture socio-demographic, lifestyle, and health-related variables. Hierarchical cluster analysis was utilized to identify patterns of multimorbidity and associated factors.

**Results:**

Among 541 participants, 66.17% had multimorbidity, defined as the presence of two or more chronic conditions, with a mean of 2.39 ± 1.71 chronic conditions per participant. Hypertension and type 2 diabetes mellitus were the most prevalent conditions, while malignant diseases were observed in only a small proportion of participants. Polypharmacy was observed in 13.9% of the study population and was significantly more frequent among men. Hierarchical cluster analysis identified two major multimorbidity clusters, one characterized predominantly by cardiometabolic and degenerative conditions and another associated with sociodemographic and lifestyle factors. Statistically significant associations were observed between multimorbidity and age, sex, education, income, and dietary patterns.

**Conclusion:**

This study highlights a high burden of multimorbidity among geriatric outpatients in South India, with hypertension and diabetes being the most prevalent. Clear gender-specific patterns and strong associations with socio-demographic and dietary factors were identified. The findings underscore the importance of individualized, gender-sensitive, and nutrition-focused strategies in the management of multimorbidity among older adults.

## 1 Introduction

Population aging is a significant global phenomenon, with the number of older adults aged 60 and above increasing from 901 million in 2015 to over 2.1 billion by 2050. The aging process presents several challenges, particularly in the field of healthcare, as the older adults population is disproportionately affected by chronic diseases. Chronic diseases are among the leading causes of death globally, accounting for 71% of all deaths, with an estimated 41 million deaths annually. Multimorbidity, defined as the coexistence of two or more chronic conditions within an individual, complicates patient care by increasing diagnostic and therapeutic complexity and is associated with poorer health outcomes, longer hospital stays, and higher healthcare costs (1). As the older adults population increases, the prevalence of chronic diseases also rises. This trend is observed in many countries and is expected to further strain healthcare systems with limited resources (2). Over the years, considerable primary care research has been conducted in developed nations, emphasizing the importance of this issue. Current estimates of multimorbidity prevalence vary, reaching as high as 39.5% in Spain and 13% in the Netherlands. Middle-income countries such as Ghana, Brazil, and South Africa report multimorbidity rates up to 38.5%. The number of individuals facing multimorbidity is likely to increase worldwide, emphasizing the need for more sustainable care models. However, a key challenge remains in identifying the most critical areas for intervention (3). By 2020, around 20% of the older adults population in China was affected by multiple chronic conditions, and this burden is expected to rise further (4).

India is projected to experience a substantial increase in its older population over the coming decades, accompanied by a rising burden of chronic diseases and multimorbidity, posing significant challenges for health systems and geriatric care delivery (LASI Wave 1, IIPS and MoHFW). In Andhra Pradesh rural areas, the prevalence of multimorbidity among older adults was found to be 28.3%, increasing from 5.8% among those aged 18–29 years to 45% among individuals aged 70 years and above. Factors contributing to multimorbidity included older age, gender, higher education levels, and greater income, possibly reflecting increased health awareness, better healthcare access, and more frequent diagnosis in these groups (5). Furthermore, a nationally representative cross-sectional study in India reported an overall multimorbidity prevalence of 7.2%, with higher rates in urban areas (9.7%) compared to rural areas (5.8%). The most frequently occurring combinations of chronic conditions were hypertension and obesity, hypertension and anemia, and obesity and anemia (6). This growing burden of chronic diseases presents a major challenge for the healthcare system, as chronic conditions account for over 70% of the total disease burden (7).

The co-occurrence of multiple chronic diseases, or multimorbidity, is associated with adverse health outcomes, including functional decline, reduced quality of life, and increased healthcare utilization. Hypertension, diabetes mellitus, arthropathies, and cardiovascular diseases are among the most commonly observed conditions in individuals with multimorbidity (8). As the number of comorbid conditions increases, polypharmacy, defined as the concurrent use of five or more medications, becomes more prevalent and has been linked to adverse outcomes such as hospitalization, drug interactions, and poor treatment adherence (8). Despite these challenges, existing healthcare guidelines largely focus on single-disease management, underscoring the need for more integrated, person-centered approaches to multimorbidity care.

Despite existing Indian studies reporting the prevalence of multimorbidity among older adults, evidence from geriatric outpatient settings in low- and middle-income countries remains limited, particularly studies that integrate comprehensive geriatric assessment with lifestyle, nutritional, and frailty indicators to identify patterns of co-occurring diseases. Understanding these patterns is important because multimorbidity is associated with increased healthcare utilization, polypharmacy, functional decline, and reduced quality of life among older adults (9). Therefore, this study aimed to assess the prevalence and patterns of multimorbidity among older adults attending a tertiary care outpatient clinic in South India and to examine its associations with sociodemographic, lifestyle, and clinical factors using comprehensive geriatric assessment and hierarchical cluster analysis.

## 2 Methods

### 2.1 Study design and data collection

The cross-sectional observational study was conducted at the Geriatric Clinic of a tertiary care hospital in South India over 2 years, from November 2022 to November 2024. The hospital serves as a major referral center for surrounding urban and rural districts, catering to a diverse population across multiple socioeconomic backgrounds. The study was approved by the Institutional Ethics Committee (IEC approval number: JSSMC/IEC/011222/01 NCT/2022–23). Patients are referred to the geriatric clinic from outpatient departments, primary health centers, and community health programs, as well as through inpatient referrals from various specialties within the hospital. This broad catchment area allows for a comprehensive representation of older adults with varying degrees of multimorbidity and functional status. A total of 541 older adults individuals were recruited using a non-probability sampling technique. The sample size was determined based on the expected prevalence of multimorbidity reported in previous studies, and all eligible participants attending the geriatric outpatient clinic during the study period were consecutively recruited. Participants were selected consecutively based on their attendance at the outpatient geriatric clinic and their willingness to participate. The study primarily included adults aged 60 years and above who attended the geriatric outpatient clinic; a small number of participants below 60 years were also enrolled during routine clinical screening. Participants below 60 years of age were retained only for descriptive clinical assessment but were not included in age-specific subgroup interpretations. Those who were inpatients or presented with acute illnesses at the time of recruitment were excluded. After obtaining informed consent, each participant underwent screening for multimorbidity, followed by a detailed Comprehensive Geriatric Assessment (CGA). The assessment and data collection were conducted using a structured pro forma. The CGA comprised multiple domains: (i) sociodemographic assessment, which collected information on age, gender, education, income, marital status, and living arrangements; (ii) environmental assessment, which evaluated housing conditions, access to healthcare, and availability of social support; (iii) medical assessment, which included a review of medical history, current diagnoses, medication review (including polypharmacy), and a brief physical examination covering vital signs and systems review. Although a full neurological examination was not conducted, cognitive function was evaluated separately; (iv) functional assessment, which used validated tools such as the Barthel Index to assess basic activities of daily living (ADLs); (v) psychosocial assessment, which included screening for depression, anxiety, and cognitive impairment using tools such as the Geriatric Depression Scale (GDS) and Mini-Mental State Examination (MMSE). In this study, the impact of multimorbidity was operationally defined using validated measures of mental health and functional status, including depressive symptoms assessed by the Geriatric Depression Scale (GDS), cognitive function assessed by the Mini-Mental State Examination (MMSE), and functional ability assessed using the Barthel Index. (vi) advance care planning, which assessed participants’ awareness and preferences regarding future care and end-of-life decisions; (vii) spiritual well-being assessment, which explored the role of faith and spiritual practices in coping and well-being. Given the relevance of oral health in frailty, a focused oral health screening was also included, though it did not constitute a full dental examination; and (viii) nutritional assessment, which collected dietary information including meal frequency, intake of vegetables, legumes, dairy products, and daily fluid consumption using a structured questionnaire based on participant self-report during the Comprehensive Geriatric Assessment.

All assessments were conducted by trained healthcare personnel, including nurses, physiotherapists, and social workers, who received specific instruction on the use of standardized tools and protocols prior to data collection. Training sessions ensured uniform understanding and application of assessment procedures, such as administering the Barthel Index for functional status, the Geriatric Depression Scale (GDS) for assessing depressive symptoms, and the Mini-Mental State Examination (MMSE) for evaluating cognitive function. Clinical parameters such as blood pressure, weight, and comorbidities were measured using standardized techniques. Regular supervision and periodic cross-checks were implemented during the data collection period to maintain inter-rater reliability and ensure accuracy and consistency across assessors.

As this was a hospital-based study using non-probability consecutive sampling, the generalizability of the findings may be limited to similar tertiary care geriatric outpatient settings; however, this approach provides clinically relevant insights into real-world multimorbidity patterns.

### 2.2 Outcome variable: multimorbidity

The primary outcome of this study was multimorbidity, defined as the presence of two or more chronic conditions (≥2) in an individual. In this study, all chronic conditions were treated as individual diagnoses and were counted equally without applying weighting for disease severity, functional impact, or interactions between co-occurring conditions. Chronic conditions were identified based on physician-diagnosed illnesses documented during the Comprehensive Geriatric Assessment and verified through medical records. The total number of chronic conditions per participant was calculated by summing all documented diagnoses included in the medical assessment. The chronic diseases considered for defining multimorbidity in this study included hypertension, type 2 diabetes mellitus, osteoarthritis, cerebrovascular accident, peptic ulcer disease, chronic obstructive pulmonary disease (COPD), chronic kidney disease, rheumatoid arthritis, ischemic heart disease, solid tumors, leukemia, and other chronic conditions reported during the clinical evaluation. Participants with two or more of these chronic conditions were categorized as having multimorbidity. Participants with incomplete or missing information for key study variables were excluded from the respective analyses.

Key variables such as multimorbidity and polypharmacy were derived from clinical assessment and medical records, with multimorbidity defined as ≥2 chronic conditions and polypharmacy defined as the concurrent use of multiple medications.

### 2.3 Covariables

The covariables consisted of socio-demographic and lifestyle factors gathered from the Comprehensive Geriatric Assessment. The socio-demographic variables included age, gender, education level, occupation, monthly income, socioeconomic class, financial dependence, and living arrangements. The lifestyle and nutritional factors comprised the number of meals consumed per day, the intake of dairy products, vegetables, legumes, or protein sources, daily fluid intake, and additional indicators such as polypharmacy, weight changes, and appetite loss. These variables were analyzed primarily as individual-level covariates in exploratory analyses, and potential interaction or mediation effects between socioeconomic and lifestyle factors were not formally examined in the present study.

### 2.4 Statistical analysis

Data are expressed as mean ± standard deviation (SD) for continuous variables and as numbers with percentages for categorical variables. To investigate the relationships between multimorbidity and various lifestyle factors, as well as their effects on mental health and frailty, both descriptive and inferential statistics were employed. Group comparisons for continuous variables were performed using the student’s t-test and one-way analysis of variance (ANOVA), while categorical variables were compared using the Chi-square (χ^2^) test. For variables not normally distributed, Spearman’s rho correlation was used to evaluate the strength and direction of associations. Lifestyle and dietary variables were categorized into predefined groups (e.g., number of meals per day, frequency of specific food intake, and levels of daily fluid consumption) for analysis.

To identify patterns of multimorbidity, hierarchical cluster analysis was performed using Ward’s minimum variance method with squared Euclidean distance as the dissimilarity measure. Ward’s method is an agglomerative hierarchical clustering technique that begins with each variable in its own cluster and progressively merges them to minimize the within-cluster sum of squares. The optimal number of clusters was determined based on dendrogram inspection. The clustering procedure included selected clinical conditions, lifestyle factors, and sociodemographic variables related to multimorbidity. Categorical variables were coded appropriately prior to analysis to enable their inclusion in the clustering procedure. The number of clusters was determined based on visual inspection of the dendrogram and the interpretability of the resulting cluster groupings. The resulting cluster structure was visualized using a dendrogram generated in IBM SPSS Statistics Version 22.0, where vertical lines represent the joining of clusters and horizontal lines indicate the level of dissimilarity between them (with longer lines reflecting greater dissimilarity). All statistical analyses were conducted using IBM SPSS Statistics Version 22.0 and Microsoft Excel 2019 (10). All analyses were exploratory and primarily based on unadjusted bivariate correlations. Additionally, interaction effects between selected covariates were explored to evaluate their potential combined influence on multimorbidity. Given the exploratory nature of the study, no formal correction for multiple comparisons was applied; therefore, the results were interpreted with caution.

### 2.5 Web graph

**Figure 1:**
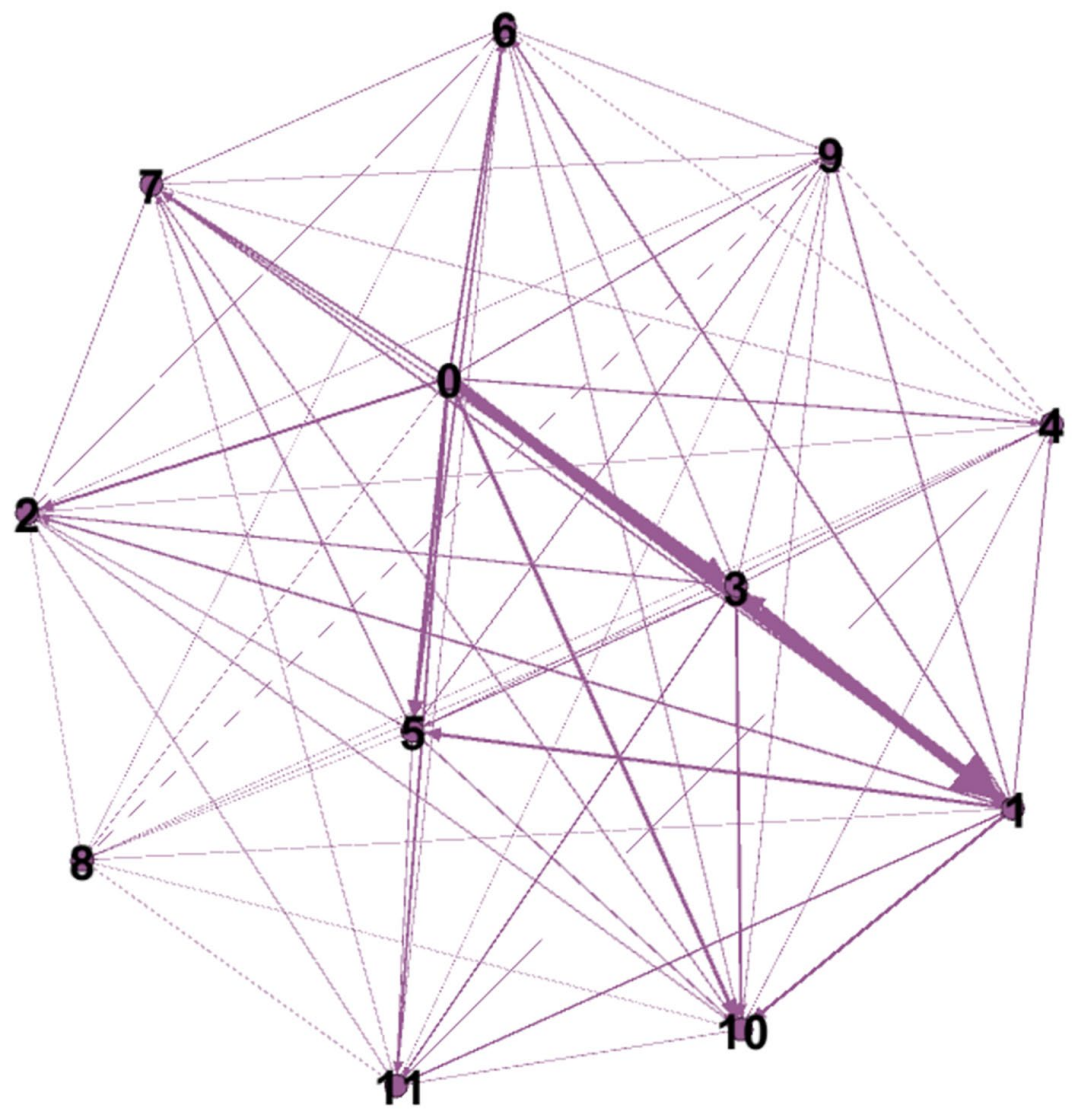
Co-occurrence web graph of comorbidities in the study population. The network displays the relationships among 12 comorbid conditions, represented by nodes labeled 0–11. Directed edges indicate the frequency of co-occurrence between pairs, with thicker edges representing stronger associations. The strongest connection was observed between nodes 0 and node labels: 0 – HTN, 1 – Type 2 DM, 2 – COPD, 3 – Osteoarthritis, 4 – IHD, 5 – CVA, 6 – RA, 7 – CKD, 8 – Leukemia, 9 – Solid tumor, 10 – Peptic ulcer, 11 – other.

Comorbidities were visualized using web graphs (11). In these graphs, each node represents a specific comorbid condition, while the edges between nodes indicate the frequency of co-occurrence between two diseases. Thick solid lines signify strong connections with a comorbidity frequency greater than 200 cases, whereas normal solid and dotted lines represent moderate and weak connections. The size of each node corresponds to the relative frequency of the individual comorbidity. For instance, Figure 1 illustrates the co-occurrence web graph of 12 comorbidities, where nodes are labeled 0–11, and the thickness of the edges reflects the strength of the co-occurrence between the corresponding comorbidities.

## 3 Results

### 3.1 Demographic and clinical profile of participants

The demographic characteristics of the study population are presented below:

The study included 541 participants, consisting of 280 males and 261 females, with an average age of 71.56 ± 7.06. Men had a higher mean age than women (72.25 ± 7.32 vs. 70.71 ± 6.71; *p* < 0.00001). As illustrated in Table 1, most participants fell within the 60 to 79 age range. There were significant differences between genders in occupations; a majority of men were retired professionals or business owners, while most women were housewives (*p* < 0.00001). Educational levels showed a disparity as well; men had higher levels of education, while women had a higher proportion of illiteracy (*p* < 0.0001). Financial dependence was higher among women (*p* < 0.00001), and men were more often in the income bracket above (*p* = 0.0288). Most of the participants resided with their spouses and children (44.2%) or with children alone (41.0%), with living arrangements differing by gender, with men more frequently residing in dual-generation households (*p* < 0.00001). Polypharmacy was noted in 13.9%, with a significantly higher occurrence among men (*p* = 0.005). The majority reported having three meals a day (88.7%) and maintained a moderate consumption of dairy, vegetables, and protein, with a significant gender difference observed in dairy intake (*p* = 0.0028). No significant variations were detected in fluid consumption, recent weight changes, or loss of appetite. Regarding frailty assessments, participants were primarily categorized as vulnerable (51.9%) or normal (25.7%), while 22.4% were classified as frail according to the Clinical Frailty Scale. Women had a higher prevalence of slightly elevated oral frailty risk (*p* = 0.019). Hypertension (64.9%) and type 2 diabetes (49.0%) were the most prevalent comorbid conditions. Cerebrovascular accidents were more frequent among men (*p* = 0.0377), while other health issues did not reveal marked gender differences.

**Table 1:** Characteristics of patients (*n* = 541), grouped by gender.

Characteristic	Overall (*n* = 541)	Men (*n* = 280)	Women (*n* = 261)	*p* value
Demographics				
Age (years) SD	71.56 ± 7.06	72.25 ± 7.32	70.71 ± 6.71	<0.05
<50	2 (0.4)	2 (0.4)	0 (0.0)	
50–59	6 (1.1)	1 (0.2)	5 (0.9)	
60–69	221 (40.9)	103 (19.0)	118 (21.8)	
70–79	229 (42.3)	125 (23.1)	104 (19.2)	
80–89	78 (14.4)	44 (8.1)	34 (6.3)	
≥90	5 (0.9)	5 (0.9)	0 (0.0)	
Occupation				
Farmer, Retired official, Receives pension, Business/Own business	283 (52.3)	255 (47.1)	28 (5.2)	<0.05
Housewife and others	252 (46.6)	21 (3.9)	231 (42.7)	<0.05
Tailor, Painter	3 (0.6)	2 (0.4)	1 (0.2)	0.60
Factory worker, Works at bakery	2 (0.4)	1 (0.2)	1 (0.2)	0.96
Journalist	1 (0.2)	1 (0.2)	0 (0.0)	0.33
Literacy status				
Professional degree	5 (0.9)	4 (0.7)	1 (0.2)	0.20
Graduate	30 (5.5)	25 (4.6)	5 (0.9)	<0.05
Intermediate	1 (0.2)	1 (0.2)	0 (0.0)	0.33
High school	80 (14.8)	56 (10.4)	24 (4.4)	<0.05
Primary school	302 (55.8)	150 (27.7)	152 (28.1)	0.27
Illiterate	123 (22.7)	44 (8.1)	79 (14.6)	<0.05
Financial status				
Dependent	408 (75.4)	170 (31.4)	238 (44.0)	<0.05
Independent	133 (24.6)	110 (20.3)	23 (4.3)	<0.05
Income monthly				
₹47,348 and above	508 (93.9)	269 (49.7)	239 (44.2)	<0.05
₹23,674–₹47,347	33 (6.1)	11 (2.0)	22 (4.1)	<0.05
Medical/life insurance				
Yes	30 (5.5)	18 (3.3)	12 (2.2)	0.35
No	511 (94.5)	262 (48.4)	249 (46.0)	0.35
Living status				
Other	1 (0.2)	0 (0.0)	1 (0.2)	0.29
Alone	26 (4.8)	9 (1.7)	17 (3.1)	0.07
Elderly care center	1 (0.2)	1 (0.2)	0 (0.0)	0.33
Old age	1 (0.2)	1 (0.2)	0 (0.0)	0.33
With children	222 (41.0)	77 (14.2)	145 (26.8)	<0.05
With sister	1 (0.2)	0 (0.0)	1 (2.0)	0.29
With spouse	50 (9.2)	34 (6.3)	16 (3.0)	<0.05
With spouse and children	239 (44.2)	158 (29.2)	81 (15.0)	<0.05
Life style				
Polypharmacy				
No	466 (88.1)	230 (42.5)	236 (43.6)	<0.05
Yes	75 (13.9)	50 (9.2)	25 (4.6)	<0.05
Number of meals taken/day				
1	3 (0.6)	3 (0.6)	0 (0.0)	0.09
2	55 (10.2)	31 (5.7)	24 (4.4)	0.47
3	480 (88.7)	244 (45.1)	236 (43.6)	0.22
4	3 (0.6)	2 (0.4)	1 (0.2)	0.60
Consumption pattern of dairy products/day				
0	32 (5.9)	15 (2.8)	17 (3.1)	0.56
1	377 (69.7)	195 (36.0)	182 (33.6)	0.98
2	129 (23.8)	69 (12.8)	60 (11.1)	<0.05
3	3 (0.6)	1 (0.2)	2 (0.4)	0.52
Consumption pattern of legumes or egg or meat/day				
0	158 (29.2)	84 (15.5)	74 (13.7)	0.67
1	294 (54.3)	147 (27.2)	147 (27.2)	0.37
2	86 (15.9)	46 (8.5)	40 (7.4)	0.72
3	2 (0.4)	2 (0.4)	0 (0.0)	0.17
4	1 (0.2)	1 (0.2)	0 (0.0)	0.33
Consumption pattern of vegetable/day				
0	20 (3.7)	10 (1.9)	10 (1.9)	0.87
1	343 (63.5)	174 (32.2)	169 (31.3)	0.52
2	175 (32.4)	93 (17.2)	82 (15.2)	0.65
3	2 (0.4)	2 (0.4)	0 (0.0)	0.17
Fluid intake (in litres/day)				
1	36 (6.7)	17 (3.2)	19 (3.5)	0.57
2	471 (87.4)	245 (45.5)	226 (41.9)	0.75
3	32 (5.9)	18 (3.3)	14 (2.6)	0.59
Weight changes in last 6 months				
No	514 (95.0)	267 (49.4)	247 (45.7)	0.70
Yes	27 (5.0)	13 (2.4)	14 (2.6)	0.70
Any loss of appetite				
No	455 (84.1)	237 (43.8)	218 (40.3)	0.72
Yes	86 (15.9)	43 (7.9)	43 (7.9)	0.72
Fraility assessment				
General fraility				
Normal	139 (25.7)	78 (14.4)	61 (11.3)	0.23
Vulnerable	281 (51.9)	137 (25.3)	144 (26.6)	0.14
Mild	84 (15.5)	42 (7.8)	42 (7.8)	0.72
Moderate	33 (6.1)	20 (3.7)	13 (2.4)	0.29
Severe	4 (0.7)	3 (0.6)	1 (0.2)	0.35
Oral fraility				
Highest risk	60 (11.1)	31 (5.7)	29 (5.4)	0.98
Lower risk	135 (25.0)	66 (12.2)	69 (12.8)	0.44
Slightly increased risk	346 (64.0)	166 (30.7)	180 (33.3)	<0.05
Co morbidity				
HTN				
Yes	351 (64.9)	175 (32.3)	176 (32.5)	0.22
No	190 (35.1)	105 (19.4)	85 (15.7)	0.22
Type 2 DM				
Yes	265 (49.0)	134 (24.8)	131 (24.2)	0.58
No	276 (51.0)	146 (27.0)	130 (24.0)	0.58
COPD				
Yes	82 (15.2)	40 (7.4)	42 (7.8)	0.55
No	459 (84.8)	240 (44.4)	219 (40.5)	0.55
Osteoarthritis				
Yes	118 (21.8)	61 (11.3)	57 (10.5)	0.99
No	423 (78.2)	219 (40.5)	204 (37.7)	0.99
IHD				
Yes	37 (6.8)	21 (3.9)	16 (3.0)	0.52
No	504 (93.2)	259 (47.9)	245 (45.3)	0.52
CVA				
Yes	107 (19.8)	65 (12.0)	42 (7.8)	<0.05
No	434 (80.2)	215 (39.7)	219 (40.5)	<0.05
RA				
Yes	66 (12.2)	34 (6.3)	32 (5.9)	0.96
No	475 (87.8)	246 (44.5)	229 (42.3)	0.96
CKD				
Yes	55 (10.2)	32 (5.9)	23 (4.3)	0.31
No	486 (89.8)	248 (45.8)	238 (44.0)	0.31
Leukemia				
Yes	7 (1.3)	4 (0.7)	3 (0.6)	0.77
No	534 (98.7)	276 (51.0)	258 (47.7)	0.77
Solid tumor				
Yes	37 (6.8)	19 (3.5)	18 (3.3)	0.95
No	504 (93.2)	261 (48.2)	243 (44.9)	0.95
Peptic ulcer				
Yes	94 (17.4)	52 (9.6)	42 (7.8)	0.44
No	447 (82.6)	228 (42.1)	219 (40.5)	0.44
Others				
Yes	79 (14.6)	40 (7.4)	39 (7.2)	0.82
No	462 (85.4)	240 (44.4)	222 (41.0)	0.82

Values are presented as numbers (percentages) for categorical variables and mean ± standard deviation for continuous variables. Percentages are calculated within each gender group unless otherwise specified. Comparisons between men and women were made using the chi-square test for categorical variables and the independent t-test for continuous variables. Statistically significant *p*-values (<0.05) indicate gender differences across variables. HTN, Hypertension; DM, Diabetes mellitus; COPD, Chronic Obstructive Pulmonary Disease; IHD, Ischemic heart disease; CVA, Cerebrovascular accident; RA, Rheumatoid arthritis; CKD, Chronic kidney disease.

### 3.2 Prevalence of multimorbidity among older adults in the study population

The lifestyle-related characteristics of the participants are summarized in this section.

Among 541 participants, 66.17% (*n* = 358) had multimorbidity, defined as the presence of two or more chronic conditions. Across all participants (*n* = 541), the mean number of chronic conditions per participant was 2.39 ± 1.71. Multiple comorbidities were commonly observed in the study population (Supplementary Table S1). Hypertension (64.9%) and type 2 diabetes mellitus (49.0%) were the most prevalent chronic conditions, followed by osteoarthritis (21.8%), cerebrovascular accidents (19.8%), peptic ulcer disease (17.4%), chronic obstructive pulmonary disease (15.2%), and chronic kidney disease (10.2%).

Less prevalent conditions included rheumatoid arthritis (12.2%), ischemic heart disease (6.8%), solid tumors (6.8%), and leukemia (1.3%), indicating that malignant conditions were present in only a small proportion of participants. Cardiometabolic and degenerative conditions accounted for the majority of reported conditions, while malignant conditions were less frequent.

### 3.3 Association of multimorbidity with socio-demographic and lifestyle factors

The relationships between multimorbidity and selected variables are presented below:

Spearman’s correlation analysis (Table 2) showed that a higher frequency of meals per day had a weak negative correlation with osteoarthritis (*r* = −0.182, *p* < 0.001) and weak negative correlations with peptic ulcer disease (*p* < 0.05). Dairy intake showed a weak positive correlation with osteoarthritis (*r* = 0.121, *p* = 0.005) and weak negative correlations with ischemic heart disease, rheumatoid arthritis, chronic kidney disease, and solid tumors (*p* < 0.05). Higher consumption of legumes, eggs, or meat showed a weak positive correlation with osteoarthritis (*r* = 0.141, *p* = 0.001) and weak negative correlations with rheumatoid arthritis, chronic kidney disease, and COPD. Vegetable intake showed a weak negative correlation with rheumatoid arthritis (*r* = −0.179, *p* < 0.001). Fluid intake did not show significant correlations with any of the chronic conditions studied.

**Table 2:** Association of multimorbidity with lifestyle factors.

Correlation	Lifestyle factors	Statistical parameter	HTN	Type 2 DM	COPD	Osteoarthritis	IHD	CVA	RA	CKD	Leukemia	Solid tumor	Peptic ulcer	Others
Spearman’s rho	Number of meals taken daily	Correlation Coefficient	0.030	0.057	−0.065	−0.182**	−0.003	0.029	−0.028	−0.028	0.037	−0.075	−0.097*	−0.099*
		Sig. (2-tailed)	0.488	0.187	0.135	0.000	0.942	0.509	0.524	0.512	0.389	0.083	0.026	0.023
	Consumption pattern of dairy products/day	Correlation Coefficient	0.048	0.043	−0.027	0.121**	−0.043	−0.130**	−0.106*	−0.094*	−0.012	−0.116**	0.047	0.044
		Sig. (2-tailed)	0.271	0.320	0.541	0.005	0.321	0.003	0.015	0.030	0.774	0.007	0.278	0.312
	Consumption pattern of legumes or egg or meat per/day	Correlation Coefficient	0.077	0.087*	−0.036	0.141**	−0.009	−0.089*	−0.148**	−0.099*	−0.024	−0.078	0.061	0.028
		Sig. (2-tailed)	0.077	0.044	0.403	0.001	0.829	0.041	0.001	0.022	0.586	0.073	0.159	0.517
	Consumption pattern of vegetables/day	Correlation Coefficient	−0.016	−0.022	−0.105*	−0.016	−0.094*	−0.110*	−0.179**	−0.109*	−0.058	−0.110*	−0.012	0.001
		Sig. (2-tailed)	0.711	0.618	0.016	0.715	0.030	0.011	0.000	0.012	0.179	0.011	0.786	0.979
	Fluid intake (in litres/day)	Correlation Coefficient	−0.004	−0.011	−0.051	−0.015	−0.057	0.024	0.057	−0.064	0.003	0.069	0.052	−0.021
		Sig. (2-tailed)	0.918	0.798	0.244	0.735	0.190	0.586	0.188	0.141	0.952	0.110	0.233	0.626

^*^Correlation is significant at the 0.05 level (2-tailed). ^**^Correlation is significant at the 0.01 level (2-tailed). HTN, Hypertension; DM, Diabetes mellitus; COPD, Chronic obstructive pulmonary disease; IHD, Ischemic heart disease; CVA, Cerebrovascular accident; RA, Rheumatoid arthritis; CKD, Chronic kidney disease.

Socio-demographic factors showed weak correlations with selected chronic conditions. Literacy showed a weak positive correlation with type 2 diabetes (*r* = 0.109, *p* = 0.012) and solid tumors (*r* = 0.113, *p* = 0.009), and a weak negative correlation with COPD (*r* = −0.119, *p* = 0.006). Monthly income and socioeconomic class showed weak correlations with type 2 diabetes (*r* = 0.107, *p* = 0.014), while living status showed a weak correlation with cerebrovascular accidents (*r* = 0.100, *p* = 0.022). Occupation did not show significant correlations with any chronic illnesses.

Chi-square analysis showed significant associations between polypharmacy and type 2 diabetes, osteoarthritis, ischemic heart disease, chronic kidney disease, and peptic ulcer disease (all *p* ≤ 0.001) (Supplementary Tables S2, S3). Weight changes and appetite loss did not show consistent significant associations with multimorbidity, with only a few weak correlations observed for COPD and peptic ulcer disease (Table 3).

**Table 3:** Association of multimorbidity with sociodemographic.

Correlation	Sociodemographic variables	Statistical parameter	HTN	Type 2 DM	COPD	Osteoarthritis	IHD	CVA	RA	CKD	Leukemia	Solid tumor	Peptic ulcer	Others
Spearman’s rho	Literacy Status	Correlation Coefficient	0.061	0.109*	−0.119**	0.020	−0.002	0.056	−0.032	0.034	0.046	0.113**	−0.005	0.053
		Sig. (2-tailed)	0.163	0.012	0.006	0.653	0.958	0.199	0.458	0.435	0.293	0.009	0.900	0.226
	Income-monthly	Correlation Coefficient	0.034	0.050	0.060	0.034	0.036	−0.018	−0.058	−0.023	0.029	−0.029	−0.055	0.034
		Sig. (2-tailed)	0.438	0.249	0.170	0.434	0.403	0.678	0.181	0.599	0.509	0.505	0.207	0.437
	Socio economic class	Correlation Coefficient	0.013	0.107*	−0.029	0.040	0.061	0.064	−0.007	0.062	0.055	0.056	−0.018	0.040
		Sig. (2-tailed)	0.760	0.014	0.501	0.356	0.157	0.141	0.873	0.153	0.205	0.195	0.686	0.357
	Living status	Correlation Coefficient	0.009	−0.035	−0.048	−0.017	0.014	0.100*	0.007	0.020	0.008	0.029	−0.045	−0.037
		Sig. (2-tailed)	0.828	0.424	0.266	0.695	0.750	0.022	0.877	0.652	0.846	0.506	0.302	0.397
	Occupation	Correlation Coefficient	−0.073	−0.028	0.001	0.023	0.050	0.070	−0.004	0.042	0.010	0.014	0.007	0.018
		Sig. (2-tailed)	0.092	0.513	0.983	0.594	0.247	0.108	0.919	0.330	0.825	0.749	0.869	0.680

*Correlation is significant at the 0.05 level (2-tailed). **Correlation is significant at the 0.01 level (2-tailed). HTN, Hypertension; DM, Diabetes mellitus; COPD, Chronic obstructive pulmonary disease; IHD, Ischemic heart disease; CVA, Cerebrovascular accident; RA, Rheumatoid arthritis; CKD, Chronic kidney disease.

### 3.4 Multimorbidity clusters and their associations with sociographic and lifestyle factors

#### 3.4.1 Multimorbidity clusters and their sociographic associations

**Figure 2:**
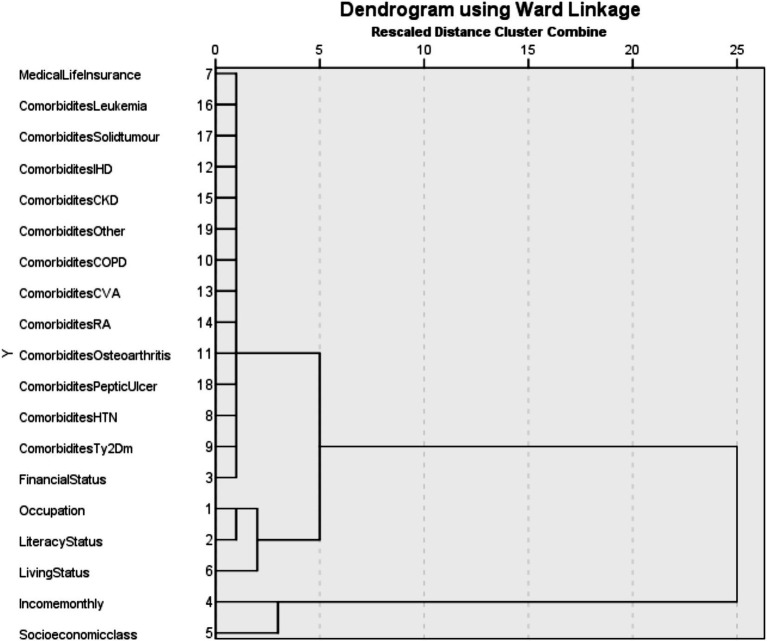
Dendrogram showing hierarchical cluster analysis of multimorbidity and sociodemographic variables using Ward’s linkage method with squared Euclidean distance.

Hierarchical cluster analysis identified two major clusters of variables (Figure 2). The first cluster comprised sociodemographic factors, including literacy status, occupation, monthly income, socioeconomic class, financial dependence, and living arrangements. The second cluster consisted predominantly of chronic health conditions such as hypertension, type 2 diabetes mellitus, osteoarthritis, peptic ulcer disease, rheumatoid arthritis, cerebrovascular accidents, chronic obstructive pulmonary disease, chronic kidney disease, ischemic heart disease, solid tumors, and leukemia. Within this disease cluster, hypertension, type 2 diabetes mellitus, peptic ulcer disease, and osteoarthritis formed a subgroup of frequently co-occurring conditions. Indicators of socioeconomic disadvantage, including low income, financial dependence, and low literacy, were closely aligned with the disease cluster.

#### 3.4.2 Multimorbidity clusters and their lifestyle associations

**Figure 3:**
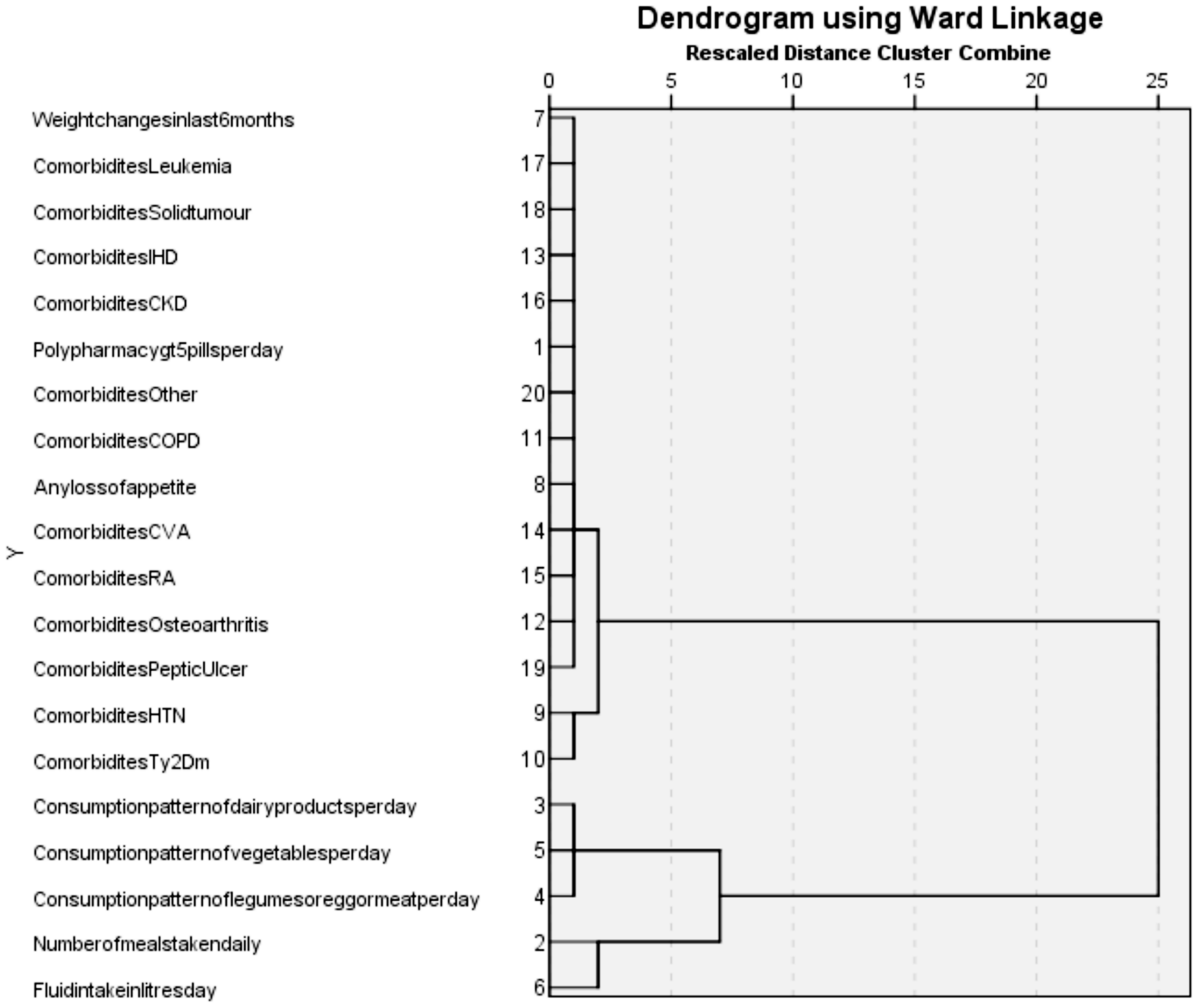
Dendrogram showing hierarchical cluster analysis of multimorbidity and lifestyle variables using Ward’s linkage method with squared Euclidean distance.

Cluster analysis examining lifestyle and nutritional factors identified two major clusters (Figure 3). One cluster grouped dietary and lifestyle variables, including number of meals per day, consumption of dairy products, vegetables, legumes or protein sources, and daily fluid intake. The second cluster comprised multiple chronic conditions, including hypertension, type 2 diabetes mellitus, osteoarthritis, peptic ulcer disease, rheumatoid arthritis, cerebrovascular accidents, chronic obstructive pulmonary disease, chronic kidney disease, ischemic heart disease, solid tumors, and leukemia. Within this disease cluster, hypertension, type 2 diabetes mellitus, peptic ulcer disease, and osteoarthritis formed a subgroup of frequently co-occurring conditions. Clinical indicators such as polypharmacy, unintentional weight change, and loss of appetite were closely aligned with the disease cluster.

### 3.5 Impact of multimorbidity on mental health and frailty

The presence of multiple chronic conditions showed correlations with mental health and frailty indicators. Rheumatoid arthritis (RA) showed a significant negative correlation with memory (*r* = −0.176, *p* < 0.01) and spiritual well-being (*r* = −0.170, *p* < 0.01). Osteoarthritis showed a moderate negative correlation with sleep duration (*r* = −0.335, *p* < 0.01) and a moderate positive correlation with general frailty (*r* = 0.299, *p* < 0.01). Ischemic heart disease (IHD) and chronic kidney disease (CKD) showed positive correlations with memory impairment (*r* = 0.162 and *r* = 0.085, respectively). Leukemia and COPD showed weak but significant correlations with cognitive impairment and frailty (Table 4).

**Table 4:** Impact of multimorbidity on mental health and frailty (Spearman’s rho correlation).

Correlation	Mental health and frailty	Statistical parameter	HTN	Type 2 DM	COPD	Osteoarthritis	IHD	CVA	RA	CKD	Leukemia	Peptic ulcer	Other
Spearman’s rho	Memory Assessment - Montreal Cognitive Assessment (MOCA)	Correlation Coefficient	−0.091*	0.022	0.002	−0.003	0.059	−0.006	−0.176**	−0.012	−0.059	0.003	−0.051
		Sig. (2-tailed)	0.035	0.607	0.963	0.937	0.174	0.886	0.000	0.781	0.173	0.951	0.237
	Spiritual well-being	Correlation Coefficient	0.032	0.007	0.030	0.083	0.002	−0.170**	−0.051	−0.059	−0.067	−0.014	−0.050
		Sig. (2-tailed)	0.462	0.875	0.480	0.052	0.956	0.000	0.240	0.170	0.117	0.752	0.248
	Memory	Correlation Coefficient	0.082	0.117**	−0.019	0.162**	0.042	0.017	0.025	0.003	0.085*	0.021	0.128**
		Sig. (2-tailed)	0.058	0.006	0.654	0.000	0.332	0.688	0.568	0.949	0.047	0.625	0.003
	Sleep (hours per day)	Correlation Coefficient	−0.081	−0.081	−0.021	−0.335**	−0.011	0.109*	0.042	−0.014	0.023	0.105*	−0.214**
		Sig. (2-tailed)	0.059	0.060	0.631	0.000	0.796	0.011	0.334	0.751	0.599	0.015	0.000
	Geriatric depression score	Correlation Coefficient	−0.013	0.006	−0.011	−0.043	−0.036	−0.017	0.006	−0.013	−0.029	−0.021	−0.048
		Sig. (2-tailed)	0.769	0.880	0.794	0.323	0.410	0.686	0.894	0.771	0.499	0.624	0.263
	General fraility assessment	Correlation Coefficient	0.006	0.045	0.049	0.299**	0.021	−0.045	0.047	0.085*	0.001	0.048	0.138**
		Sig. (2-tailed)	0.882	0.300	0.250	0.000	0.621	0.297	0.271	0.047	0.983	0.264	0.001
	Oral fraility index	Correlation Coefficient	0.053	0.023	0.011	0.017	−0.064	−0.009	0.048	−0.079	−0.046	−0.075	−0.064
		Sig. (2-tailed)	0.221	0.588	0.801	0.688	0.136	0.829	0.264	0.065	0.280	0.083	0.140

*Correlation is significant at the 0.05 level (2-tailed). **Correlation is significant at the 0.01 level (2-tailed).

### 3.6 Web graph

A web graph was used to visualize the co-occurrence of comorbidities within the study population (Figure 1). The strongest connection was observed between hypertension and type 2 diabetes mellitus (265 cases), followed by hypertension and osteoarthritis (118 cases), and hypertension and chronic obstructive pulmonary disease (COPD) (82 cases). Other frequently co-occurring conditions included type 2 diabetes mellitus and osteoarthritis (118 cases), and type 2 diabetes mellitus and COPD (82 cases). Conditions such as peptic ulcer disease, rheumatoid arthritis (RA), and chronic kidney disease (CKD) were also interconnected with other chronic conditions.

Overall, although multiple statistically significant associations were observed, most effect sizes were small and correlations were weak. Therefore, the findings should be interpreted in terms of broader patterns rather than isolated statistically significant results.

## 4 Discussion

This study aimed to assess the prevalence and patterns of multimorbidity among older adults attending an outpatient clinic in South India and to examine its associations with sociodemographic, lifestyle, nutritional, and clinical factors. Using a comprehensive geriatric assessment and cluster analysis, we sought to identify vulnerable groups and common disease groupings contributing to multimorbidity, as well as its relationship with frailty and mental health indicators. Our findings demonstrate that multimorbidity is highly prevalent among geriatric outpatients and is closely linked with sociodemographic vulnerability, lifestyle factors, and indicators of functional decline. To our knowledge, this is among the first hospital-based studies from India to integrate comprehensive geriatric assessment, detailed nutritional and lifestyle profiling, frailty indicators, and hierarchical cluster analysis to derive clinically interpretable multimorbidity patterns among older adults. The study revealed a high prevalence of multimorbidity, with hypertension and type 2 diabetes emerging as the most common chronic conditions. In the present study, multimorbidity was commonly observed among older adults, indicating a substantial burden of chronic diseases in the study population. Similar findings have been reported in previous studies, in which multimorbidity prevalence among older adults populations ranged from 40–70%, depending on population characteristics and healthcare settings. Older age, male gender, low education, and financial dependency were associated with higher multimorbidity in unadjusted analyses. These findings are consistent with previous epidemiological studies that have identified advancing age, socioeconomic disadvantage, and limited access to healthcare as key determinants contributing to the accumulation of multiple chronic conditions in later life. Hierarchical clustering revealed two major patterns: one linking multimorbidity with sociodemographic vulnerability, and another highlighting the co-occurrence of chronic diseases alongside lifestyle and nutritional factors. Polypharmacy, frailty, and cognitive concerns were more common among individuals with multiple chronic conditions. This clustering suggests that multimorbidity in older adults is not only driven by biological ageing but is also influenced by behavioral and nutritional determinants that may be modifiable through targeted interventions. Notably, healthier dietary patterns, particularly higher vegetable and legume intake, showed inverse associations with certain chronic diseases.

A notable finding of this study is the clustering of multimorbidity with specific lifestyle and nutritional factors. For instance, the inverse association between vegetable intake and conditions such as rheumatoid arthritis and chronic kidney disease is consistent with emerging evidence on the anti-inflammatory potential of plant-based diets (12). Recent studies have increasingly emphasized the combined role of dietary patterns, socioeconomic status, and lifestyle behaviours in shaping the risk of chronic diseases and multimorbidity. Dolui et al. (13), using nationally representative data from India (NFHS-5), reported that demographic and socioeconomic factors such as age, education, and economic status significantly influence the prevalence and distribution of multimorbidity among adult men. These findings highlight the importance of addressing social determinants along with modifiable behavioral risk factors for effective prevention and management of chronic conditions. In a related study, Dolui et al. (14) demonstrated that dietary diversity is significantly associated with non-communicable diseases, where inadequate or imbalanced diets may contribute to an increased risk of NCDs, indicating the need for improved nutritional practices. Additionally, Cho et al. (15) identified key factors associated with multimorbidity among older adults, highlighting the complex interaction between aging, existing health conditions, and lifestyle-related factors. This highlights the importance of early identification of individuals at risk and improved management of multiple chronic conditions. Furthermore, Joy et al. (16) reported that non-communicable disease-related multimorbidity is associated with a higher healthcare burden and increased likelihood of catastrophic health expenditure at the household level. Their findings emphasize the need for integrated and public health strategies to reduce both the clinical and economic burden of chronic diseases. Overall, these findings support the growing evidence that dietary habits, along with socioeconomic and lifestyle factors, play a critical role in the development and progression of chronic diseases. However, the associations observed in the present study were relatively modest in magnitude and should be interpreted with caution. These findings should be considered exploratory and hypothesis-generating rather than indicative of causal relationships.

The observed association between multimorbidity and polypharmacy, particularly among men, reinforces concerns regarding the complexity of managing multiple chronic conditions in settings with fragmented care and limited medication review mechanisms (17). The overall prevalence and disease patterns observed are consistent with prior reports from low- and middle-income countries (5), with hypertension and diabetes frequently forming the core of multimorbidity clusters (8). Social determinants such as low education, limited income, and financial dependence remain important contributors, likely reflecting disparities in health literacy, access to care, and living conditions (18).

From a clinical and health systems perspective, these findings underscore the importance of integrating social and lifestyle factors into the management of multimorbidity. The clustering of chronic diseases with nutritional indicators may suggest a potential role for nutrition-sensitive and person-centered interventions; however, further longitudinal and interventional studies are required to establish causal relationships. Furthermore, the association with polypharmacy highlights the need for routine medication reconciliation and coordinated care planning. Strengthening multidisciplinary geriatric care models that include nutritional counseling, medication review, and lifestyle modification may help reduce the progression of multimorbidity and improve functional outcomes among older adults. These observations align with the World Health Organization’s Integrated Care for Older People (ICOPE) framework, which emphasizes comprehensive geriatric assessment, functional ability, and coordinated multidisciplinary care. Such approaches are particularly relevant in resource-limited settings, where optimizing functional outcomes and preventing avoidable complications can improve care efficiency and quality of life for older adults.

A key limitation of this study is the use of non-probability consecutive sampling and recruitment from a single tertiary care outpatient clinic, which may introduce selection bias and limit the generalizability of the findings to community-based or rural populations. In addition, multimorbidity in this study was defined using a simple count of chronic conditions without incorporating disease severity, functional impact, or interaction between co-occurring conditions, which may oversimplify the clinical complexity of multimorbidity. However, the hospital serves a wide catchment area, and the inclusion of unselected geriatric outpatients enhances the relevance of the findings to similar clinical settings. The cross-sectional design of the study limits the ability to establish temporal relationships and causal inferences between variables. Therefore, the observed associations between dietary patterns, multimorbidity, frailty, and mental health indicators should be interpreted with caution. Reverse causality is also possible, as individuals diagnosed with chronic conditions may modify their dietary habits or lifestyle behaviours following disease onset. In addition, the analysis relied primarily on unadjusted bivariate associations, and multivariable adjustment for potential confounding factors such as age, sex, education, and income was not performed. Additionally, socioeconomic variables such as education, income, and financial dependence were analysed primarily as individual-level characteristics, and the study did not examine potential interactions or structural pathways (e.g., gender and socioeconomic status) that may influence multimorbidity patterns. As these variables are known determinants of multimorbidity, the observed associations should be interpreted with caution, as residual confounding may influence the reported relationships. As key covariates such as age, sex, and socioeconomic status are associated with both exposure variables and health outcomes, the absence of adjustment may have resulted in either overestimation or underestimation of the observed associations. Furthermore, although hierarchical cluster analysis was used to identify patterns of multimorbidity, formal validation procedures such as cluster stability testing or sensitivity analyses were not conducted. Therefore, the identified clusters should be interpreted as exploratory and may require confirmation in future studies. Dietary patterns, meal frequency, and fluid intake were based on self-reported information and participants’ recall, which may be subject to recall and social desirability bias. In addition, dietary data were not collected using validated food frequency questionnaires or portion size estimations, which may increase the possibility of measurement error and misclassification.

Despite these limitations, the study is strengthened by a robust sample size, comprehensive geriatric assessment, and inclusion of diverse health, lifestyle, and socioeconomic variables. The application of cluster analysis enabled the identification of clinically meaningful multimorbidity patterns. Future research should focus on longitudinal studies to clarify causal pathways and evaluate the effectiveness of nutrition-sensitive interventions. Prospective studies incorporating multivariable modeling and biomarker-based assessments may further improve understanding of the complex pathways linking nutrition, lifestyle, and multimorbidity. Further exploration of gut microbiome dysbiosis in relation to renal, metabolic, and cognitive multimorbidity is also warranted, given emerging evidence supporting dietary modulation through fiber, probiotics, and legumes (19). Digital health strategies, including remote monitoring and electronic medication management, may further support safe polypharmacy management and adherence among older adults, particularly those who are homebound or living in underserved areas (20).

## 5 Conclusion

This study highlights the high prevalence and complex patterns of multimorbidity among older adults attending a tertiary care outpatient clinic in South India. Hypertension and type 2 diabetes were the most prevalent conditions, with notable gender differences in comorbidity profiles. Sociodemographic characteristics and lifestyle factors were associated with multimorbidity risk, and cluster analysis revealed distinct patterns underscoring the interplay between social vulnerability and chronic disease accumulation. The observed links between multimorbidity, polypharmacy, frailty, and mental health indicators emphasize the need for integrated, person-centered geriatric care. These findings provide important evidence to support the incorporation of comprehensive geriatric assessment, nutritional screening, frailty evaluation, and coordinated medication review into routine clinical practice. Longitudinal and interventional studies are needed to strengthen causal inference and guide evidence-based strategies for managing multimorbidity among older adults in India.

## Data Availability

The original contributions presented in the study are included in the article/Supplementary material, further inquiries can be directed to the corresponding authors.
