# Supplementary material for: Cross-sectional observational study on prevalence and pattern of multimorbidity and its impact on geriatric outpatients in a south Indian tertiary hospital
Source: Front Public Health. 2026 May 13;14:1778787. doi: 10.3389/fpubh.2026.1778787 (PMC13212225; doi:10.3389/fpubh.2026.1778787)
Supplement: Supplementary file 1 [file Table_1.DOCX]

**Supplementary tables**

**Table 1**. Distribution of comorbidities by gender in the study population (n = 541).

| Comorbidity | Male (%) | Female (%) |
| --- | --- | --- |
| HTN | 35.5 | 64.5 |
| COPD | 85.0 | 15.0 |
| Osteoarthritis | 78.2 | 21.8 |
| IHD | 93.1 | 6.9 |
| CVA | 80.3 | 19.7 |
| RA | 88.2 | 11.8 |
| CKD | 88.9 | 10.1 |
| Leukemia | 98.7 | 1.3 |
| Solid tumor | 93.2 | 6.8 |
| Petic ulcer | 82.6 | 14.4 |
| Other | 85.6 | 14.4 |

HTN – Hypertension; DM – Diabetes Mellitus; COPD – Chronic Obstructive Pulmonary Disease; IHD – Ischemic Heart Disease; CVA – Cerebrovascular Accident; RA – Rheumatoid Arthritis; CKD – Chronic Kidney Disease.

**Table 2**. Association between multimorbidity and lifestyle factors (Chi-square analysis).

| Comorbidity | Polypharmacy | Weight changes in last 6 months | Any loss of appetite |
| --- | --- | --- | --- |
| HTN | 0.012 | 0.832 | 0.45 |
| Type 2 DM | 0.000 | 0.685 | 0.191 |
| COPD | 0.020 | 0.153 | 0.409 |
| Osteoarthritis | 0.000 | 1.000 | 0.150 |
| IHD | 0.000 | 1.000 | 0.489 |
| CVA | 0.753 | 0.606 | 0.882 |
| RA | 1.000 | 0.756 | 0.582 |
| CKD | 0.000 | 0.498 | 1.000 |
| Leukemia | 0.055 | 0.287 | 1.000 |
| Solid tumour | 0.309 | 0.682 | 0.340 |
| Peptic ulcer | 0.001 | 0.175 | 0.161 |
| Others | 0.002 | 0.387 | 0.612 |

HTN – Hypertension; DM – Diabetes Mellitus; COPD – Chronic Obstructive Pulmonary Disease; IHD – Ischemic Heart Disease; CVA – Cerebrovascular Accident; RA – Rheumatoid Arthritis; CKD – Chronic Kidney Disease.

**Table 3.** Association between multimorbidity and sociodemographic factors (Chi-square analysis).

| Comorbidity | Medical life insurance |
| --- | --- |
| HTN | 0.840 |
| Type 2 DM | 0.699 |
| COPD | 0.286 |
| Osteoarthritis | 0.814 |
| IHD | 0.433 |
| CVA | 1.000 |
| RA | 0.560 |
| CKD | 0.513 |
| Leukemia | 1.000 |
| Solid tumour | 0.425 |
| Peptic ulcer | 0.801 |
| Others | 1.000 |

HTN – Hypertension; DM – Diabetes Mellitus; COPD – Chronic Obstructive Pulmonary Disease; IHD – Ischemic Heart Disease; CVA – Cerebrovascular Accident; RA – Rheumatoid Arthritis; CKD – Chronic Kidney Disease.
